# High burden of viral respiratory co-infections in a cohort of children with suspected pulmonary tuberculosis

**DOI:** 10.1186/s12879-020-05653-9

**Published:** 2020-12-04

**Authors:** M. M. van der Zalm, E. Walters, M. Claassen, M. Palmer, J. A. Seddon, A.M. Demers, M. L. Shaw, E. D. McCollum, G. U. van Zyl, A. C. Hesseling

**Affiliations:** 1 Desmond Tutu TB Centre, Department of Paediatrics and Child Health, Faculty of Medicine and Health Sciences, Stellenbosch University, Cape Town, South Africa; 2 Department of Paediatrics, Great North Children’s Hospital, Newcastle-Upon-Tyne Health Trust, Newcastle upon Tyne, UK; 3 Division of Medical Virology, Faculty of Medicine and Health Sciences, Stellenbosch University and National Health Laboratory Service, Tygerberg Hospital, Cape Town, South Africa; 4 Department of Infectious Diseases, Imperial College London, London, UK; 5 Department of Medical Biosciences, University of the Western Cape, Cape Town, South Africa; 6 Icahn School of Medicine at Mount Sinai, New York, NY USA; 7 Eudowood Division of Pediatric Respiratory Sciences, School of Medicine, Johns Hopkins University, Baltimore, USA; 8 Global Program in Respiratory Sciences, Department of Pediatrics, Johns Hopkins University, Baltimore, MD USA; 9 Health Systems Program, Department of International Health, Bloomberg School of Public Health, Johns Hopkins University, Baltimore, MD USA

**Keywords:** Respiratory viruses, pulmonary tuberculosis, Paediatric

## Abstract

**Background:**

The presentation of pulmonary tuberculosis (PTB) in young children is often clinically indistinguishable from other common respiratory illnesses, which are frequently infections of viral aetiology. As little is known about the role of viruses in children with PTB, we investigated the prevalence of respiratory viruses in children with suspected PTB at presentation and follow-up.

**Methods:**

In an observational cohort study, children < 13 years were routinely investigated for suspected PTB in Cape Town, South Africa between December 2015 and September 2017 and followed up for 24 weeks. Nasopharyngeal aspirates (NPAs) were tested for respiratory viruses using multiplex PCR at enrolment, week 4 and 8.

**Results:**

Seventy-three children were enrolled [median age 22.0 months; (interquartile range 10.0–48.0); 56.2% male and 17.8% HIV-infected. Anti-tuberculosis treatment was initiated in 54.8%; of these 50.0% had bacteriologically confirmed TB. At enrolment, ≥1 virus were detected in 95.9% (70/73) children; most commonly human rhinovirus (HRV) (74.0%). HRV was more frequently detected in TB cases (85%) compared to ill controls (60.6%) (*p* = 0.02). Multiple viruses were detected in 71.2% of all children; 80% of TB cases and 60.6% of ill controls (*p* = 0.07). At follow-up, ≥1 respiratory virus was detected in 92.2% (47/51) at week 4, and 94.2% (49/52) at week 8.

**Conclusions:**

We found a high prevalence of viral respiratory co-infections in children investigated for PTB, irrespective of final PTB diagnosis, which remained high during follow up. Future work should include investigating the whole respiratory ecosystem in combination with pathogen- specific immune responses.

## Background

An estimated 1 million children < 15 years develop tuberculosis (TB) every year [1]. Pulmonary TB (PTB) contributes to approximately 75% of the TB disease burden in children, and is difficult to confirm given the paucibacillary nature of the disease and challenges in obtaining good quality respiratory samples in young children [2]. Moreover, the clinical diagnosis of PTB in children is frequently complicated by non-specific clinical presentations, which overlap with other common respiratory illnesses [3]. This is especially true in young children living in developing countries, particularly in settings where human immunodeficiency virus (HIV) is endemic [4].

There are limited data available on the prevalence of respiratory virus co-infection in children with suspected PTB [5], while children under 5 years of age carry the highest burden of not only TB, but also of other acute non-TB respiratory tract infections [6, 7]. After careful clinical investigation and follow up, many children that present with symptoms suggestive of PTB are found to have diagnoses other than TB. This group needs to be better characterised to help clinical decision making and reduce unnecessary treatment. Respiratory virus infections likely play a crucial role in the aetiology of disease in this group of children [8]. In addition, viruses could play a role in the inception, presentation, and disease progression of children with PTB, by disrupting the immunological response of the host. Alternatively, TB disease might facilitate viral co-infection which could in turn lead to an altered TB disease presentation or more severe TB disease [9]. There is a clear need to improve our current understanding of the role of respiratory co-infections in the interaction between the developing immune system and *Mycobacterium tuberculosis* (M.tb).

In order to advance our understanding of the interaction between respiratory viruses and PTB in children, we performed a preliminary study to explore the prevalence of viral respiratory co-infections in children with TB symptoms (including children ultimately diagnosed with and without PTB) at presentation and during follow-up.

## Methods

### Cohort recruitment and procedures

This analysis was part of a larger hospital-based observational cohort. Study methods have been previously described [10]. In brief, symptomatic children < 13 years of age, routinely presenting to two public hospitals with a history and well-characterized symptoms of suspected PTB [10, 11], were consecutively enrolled from December 2015 until September 2017. We also considered that TB may present acutely, especially in young children, and included children with any duration of cough, if ≥1 of the following were present: 1) exposure to an identified TB source case in the past 12 months, 2) positive tuberculin skin test (TST) if previously negative or unknown, or 3) a chest radiograph (CXR) suggestive of TB as assessed by the study clinician. We collected at least 3 respiratory specimens for smear microscopy, Xpert® MTB/RIF, and liquid culture at enrolment. Specimens were processed at the National Health Laboratory Service, (NHLS) Tygerberg Hospital following standard protocols. Other investigations included HIV testing, TST (Mantoux, 2 Tuberculin Units PPD RT-23, Statens Serum Institute, Copenhagen) and a digital CXR (antero-posterior and lateral), evaluated by two independent experts or a third reader in order to reach consensus [12]. Follow-up visits were done 4, 8 and 24 weeks after enrolment or start of TB treatment and included sampling for respiratory viruses.

### Virus detection

Nasopharyngeal aspirates (NPAs) were collected in a sterile container by nasopharyngeal suctioning with a mucus extractor (Lasec, South Africa) at enrolment, 4 and 8 weeks later. Samples were transported in a cooler box to the reference NHLS Medical Virology Laboratory within 4 h. Commercially available virus transport medium (Davies Diagnostics, Grenada Spain) was added in the laboratory. Total nucleic acid extraction was done with the NUCLISENS® easyMAG® (bioMérieux, Marcy l’Etoile, France). Viruses were identified using a commercially available multiplex PCR (Anyplex™ II, RV16, Seegene) which includes 16 viruses considered clinically and epidemiologically relevant. These were Adenovirus (AdV), Influenza A/B virus (InfV A/B), Parainfluenzavirus 1–4 (PIV 1–4), human Rhinovirus A/B/C (HRV A/B/C), Respiratory syncytial virus A and B (RSV A/B), human Bocavirus 1–4 (HBoV 1–4), human Metapneumovirus (hMPV), human Coronavirus 229E, NL63, OC43 (HCoV 229E/NL63/OC43), and human Enterovirus (HEV). The standard PCR cycle thresholds were used according the manufacturers recommendations.

At baseline, samples were analysed in real-time, while follow up samples were analysed after storage at − 80 °C for 2–3 years.

### Classification of TB cases

Attending ward clinicians (and not the study team) were responsible for TB treatment decisions, according to national TB guidelines, and all TB test results were available to them. Children were followed for 24 weeks (6 months) or until TB treatment completion. All children were retrospectively classified according to international consensus clinical case definitions for paediatric PTB [8]; “confirmed TB”, “unconfirmed TB” and “unlikely TB”. Final categories were assigned after follow-up, allowing for assessment of treatment response and review of culture results. Children classified as “unlikely TB” are referred to as “ill controls” for analysis purposes. Ill controls were initially investigated for suspected PTB, however an alternative diagnosis was made and symptoms improved without TB treatment.

### Statistical analysis

Analyses were performed using SPSS Inc. (2001 Chicago USA version 26). Pearson’s Chi- squared test was used to compare the prevalence of viral pathogens between TB cases (confirmed and unconfirmed) and ill controls at enrolment. The Yates continuity correction was used if the expected cell size was less than 5.

## Results

Seventy-three children were enrolled, median age 22 months (interquartile range (IQR) 10.0–48.0); 56.2% were male and 17.8% were HIV-infected (Table 1). TB treatment was started in 54.8%; of which 50.0% were confirmed. Children with confirmed TB had more frequent documented exposure to TB, a positive TST result and a CXR suggestive of PTB compared to unconfirmed TB cases and ill controls. The clinical presentation was similar for all three groups (confirmed TB, unconfirmed TB, and ill controls).

**Table 1:** General characteristics of children investigated for pulmonary TB (*n* = 73)

Demographics	All (***n*** = 73; 100)	Ill controls (***n*** = 33; 45.2)	Confirmed TB (***n*** = 20; 27.4)	Unconfirmed TB (***n*** = 20; 27.4)	***P***-value
Male	41 (56.2)	21 (63.6)	10 (50.0)	10 (50.0)	0.57
Age (months)	22.0 (10.0–48.0)	22.0 (7.5–48.0)	18.0 (8.3–58.0)	22.5 (15.8–40.0)	0.66
Mixed Race	48 (65.8)	21 (63.6)	15 (75.0)	12 (60.0)	0.66
HIV exposed^a^	16 (21.9)	6 (18.2)	6 (30.0)	4 (20.0)	0.59
HIV infected	13 (17.8)	6 (18.2)	3 (15.0)	4 (20.0)	1.0
Household size	5.0 (4.0–7.0)	5.0 (4.0–7.5)	6.0 (4.0–7.8)	5.0 (3.3–7.0)	0.84
Documented TB exposure	35 (47.9)	11 (33.3)	15 (75.0)	9 (45.0)	0.01
Previous TB treatment	9 (12.3)	5 (15.2)	2 (10.0)	2 (10.0)	0.82
Positive TST^b^	23 (31.5)	4 (12.1)	12 (60.0)	7 (35.0)	< 0.01
**Presenting signs and symptoms**					
Cough Duration (days)	68 (93.2) 14 (7–42)	31 (93.9) 14 (7–42)	18 (90.0) 14 (7–26)	19 (95.0) 14 (7–60)	0.86 0.78
Wheeze Duration (days)	31 (42.5) 1 (1–2)	13 (39.4) 1 (1–2)	10 (50.0) 1 (1–2)	8 (40.0) 2.5 (1–3)	0.76 0.22
Fever Duration (days)	39 (53.4) 4 (3–21)	18 (54.5) 4 (3–11)	12 (60.0) 4 (2–28)	9 (45.0) 7 (3–53)	0.63 0.73
Lack of appetite	39 (53.4)	15 (45.5)	12 (60.0)	12 (60.0)	0.48
Lethargy	26 (35.6)	11 (33.3)	9 (45.0)	6 (30.0)	0.67
Poor growth^c^	37 (50.7)	15 (45.5)	10 (50.0)	12 (60.0)	0.36
Recent antibiotics	36 (49.3)	15 (45.5)	9 (45.0)	12 (60.0)	0.66
CXR suggestive of TB^d^	31 (42.5)	7 (21.2)	16 (80.0)	8 (40.0)	< 0.01

*TST* Tuberculin skin test, *CXR* Chest x-ray

Numbers are presented with percentages in brackets. Median values are presented with interquartile range (IQR). *P* values are calculated using Chi square test for dichotomous variables and Kruskal-Wallis for continuous variables

^a^*n* = 72 due to missing data. ^b^*n* = 63 due to missing data. ^c^*n* = 65 due to missing data. ^d^*n* = 72 due to poor quality of the CXR

**Fig. 1:**
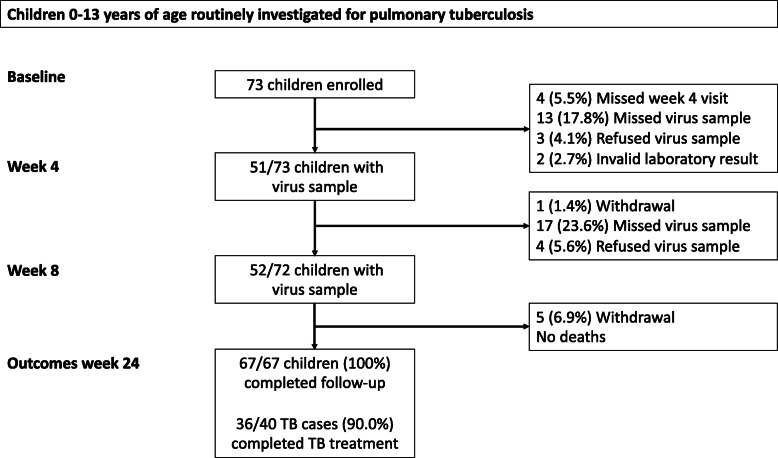
Flowchart of study participants, viral sampling and study outcomes. Flowchart of study participants from baseline to end of the study (week 24)

All 73 children had NPAs collected at enrolment, 69.9% (51/73) at week 4 and 71.2% (52/73) at week 8. Forty-three (58.9%) children had samples collected at all three time points. See Fig. 1 for flowchart of the study participants and study outcomes. There were no deaths reported; 67/ 73 (91.8%) children completed 6 months study follow-up. Thirty-six of 40 TB cases (90.0%) completed TB treatment and 4 of the TB cases withdrew before the end of the study.

At baseline, one or more viruses were detected in 95.9% (70/73) of children (Table 2). HRV was the most common, detected in 74.0% (54/73) of children, in 85% (34/40) of TB cases and in 60.6% (20/33) of ill controls, (*p* = 0.02). AdV was the second most detected virus, identified in 54.8% (40/73) of children, in 60% (24/40) of TB cases, and in 48.5% (16/33) of ill controls (*p* = 0.33). A single virus was detected at enrolment in 24.7% (18/73) of children; in 15% (6/40) of TB cases and in 36.4% (12/33) of ill controls (*p* = 0.04). Multiple viruses were detected in 71.2% (52/73) of children; 80% (32/40) in TB cases and 60.6% (20/33) of ill controls (*p* = 0.07).

**Table 2:** Detection of viruses in all children with suspected TB at baseline, in TB cases (confirmed and unconfirmed) and in ill controls (*n* = 73)

	All (***n*** = 73; 100)	TB cases (***n*** = 40, 54,8)	Ill controls (***n*** = 33; 45.2)	***P*** Value
No virus detected	3/73 (4.1)	2/40 (5.0)	1/33 (3.0)	1.00
Single virus	18/73 (24.7)	6/40 (15.0)	12/33 (36.4)	**0.04**
≥ 1 virus	70/73 (95.9)	38/40 (95.0)	32/33 (97.0)	1.00
≥ 2 viruses	52/73 (71.2)	32/40 (80.0)	20/33 (60.6)	**0.07**
≥ 3 viruses	41/73 (56.2)	27/40 (67.5)	14/33 (42.4)	**0.03**
Human Rhinovirus	54/73 (74.0)	34/40 (85.0)	20/33 (60.6)	**0.02**
Enterovirus	41/73 (56.2)	25/40 (62.5)	16/33 (48.5)	0.23
Respiratory syncytial virus (A/B)	9/73 (12.3)	5/40 (12.5)	4/33 (12.1)	1.00
Adenovirus	40/73 (54.8)	24/40 (60.0)	16/33 (48.5)	0.33
Coronavirus (229E, OC43, NL63)	9/73 (12.3)	7/40 (17.5)	2/33 (6.1)	0.17
Bocavirus	20/73 (27.4)	14/40 (35.0)	6/33 (18.2)	0.11
Influenza (A/B)	4/73 (5.5)	3/40 (7.5)	1/33 (3.0)	0.62
Parainfluenza virus (1–4)	17/73 (23.3)	11/40 (27.5)	6/33 (18.2)	0.35
Human metapneumovirus	1/73 (1.4)	1/40 (2.5)	0	1.00

Chi-square was used to detect differences between TB cases (confirmed and unconfirmed) and ill controls. The Yates continuity correction was used if the expected cell size was less than 5

Respiratory syncytial virus (RSV) A was detected in 3 samples and RSV B in 7 samples. One child had simultaneous detection of RSV A and B. Influenza A and B were simultaneously detected in 2 different samples (from different children or the same child). Parainfluenza virus (PIV) 1 and 2 were detected in 5 samples (two samples had simultaneous detection of PIV1&2), PIV 3 was not detected at all and PIV 4 was detected in 9 samples

**Fig. 2:**
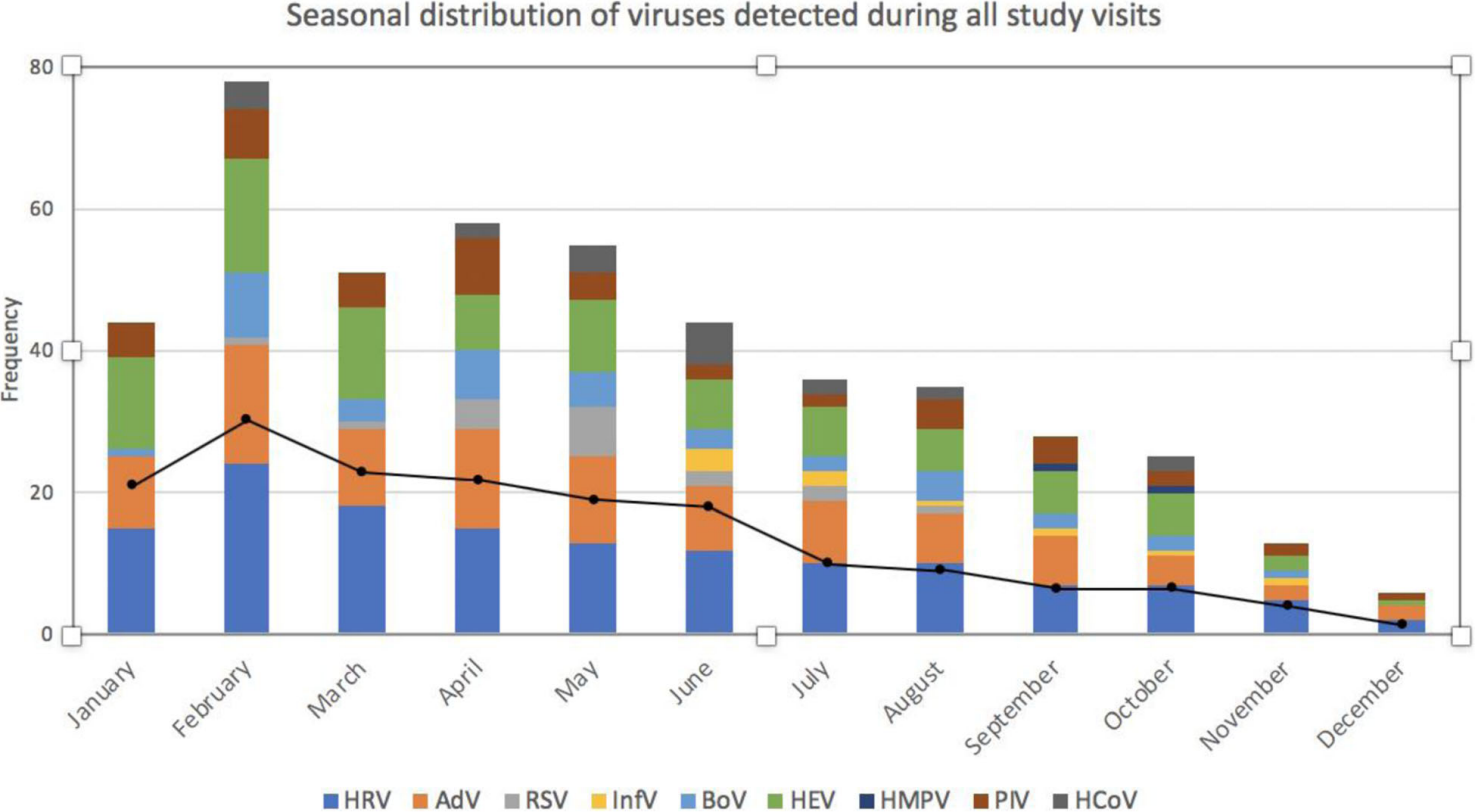
Seasonal distribution of viruses detected in children with suspected pulmonary TB. Human Rhinovirus (HRV, includes A/B/C), Adenovirus (AdV), Respiratory syncytial virus (RSV, includes A/B), Influenza virus (InfV, includes A/B), human Bocavirus (HBoV, includes 1–4), human Enterovirus (HEV), human Metapneumovirus (hMPV), Parainfluenza virus (PIV, includes 1–4) and human Coronavirus (HCoV, includes 229E/NL63/OC43). The seasonal distribution of the respiratory viruses detected in samples combined from enrolment and follow-up (TB cases and ill controls). The black line represents the number of samples collected during that month. The samples were collected from December 2015 until September 2017, therefore more samples were collected in the months January through September

Figure 2 shows the distribution of viruses detected in all samples at baseline and follow-up visits through a calendar year. Most viruses were detected throughout the year with some minor seasonal variation, while influenza virus was only detected from June to November, and RSV was detected from February to August, peaking in May.

Table 3 presents data on viruses detected at follow-up, 4 and 8 weeks after enrolment. Overall, the prevalence of viruses detected remained similar at follow up compared to baseline. One or more viruses were detected in 95.9% (70/73) children at enrolment, with 92.2% (47/51) children positive at week 4 and 94.2% (47/51) at week 8. HRV, HEV and AdV were the most detected viruses during follow up for both TB cases and ill controls.

**Table 3:** Detection of viruses during follow up at weeks 4 (*n* = 51) and 8 (*n* = 52) in children with suspected pulmonary TB

	All children		TB Cases		Ill controls	
	Week 4 ***n*** = 51	Week 8 ***n*** = 52	Week 4 ***n*** = 26	Week 8 ***n*** = 27	Week 4 ***n*** = 25	Week 8 ***n*** = 25
No virus detected	4 (7.8)	3 (5.8)	1 (3.8)	2 (7.4)	2 (8.0)	1 (4.0)
Single virus	7 (13.7)	9 (17.3)	2 (7.7)	4 (14.8)	5 (20.0)	5 (20.0)
≥ 1 virus	47 (92.2)	49 (94.2)	25 (96.2)	25 (92.6)	23 (92.0)	24 (96.0)
≥ 2 viruses	41 (80.4)	40 (76.9)	23 (88.5)	21 (77.8)	18 (72.0)	19 (76.0)
≥ 3 viruses	30 (58.8)	32 (61.5)	16 (61.5)	18 (66.7)	14 (56.0)	14 (56.0)
Human Rhinovirus	43 (84.3)	43 (82.7)	22 (84.6)	20 (74.1)	21 (84.0)	23 (92.0)
Enterovirus	26 (51.0)	29 (55.8)	14 (53.8)	17 (63.0)	12 (48.0)	12 (48.0)
Respiratory syncytial virus (A/B)	6 (11.8)	3 (5.8)	4 (15.4)	4 (14.8)	2 (8.0)	1 (4.0)
Adenovirus	33 (64.7)	31 (59.6)	17 (65.4)	15 (55.6)	16 (64.0)	16 (64.0)
Coronavirus (229E, OC43, NL63)	4 (7.8)	9 (17.3)	3 (11.5)	8 (29.6)	1 (4.0)	1 (4.0)
Bocavirus	13 (24.5)	16 (30.8)	7 (26.9)	9 (33.3)	6 (24.0)	7 (28.0)
Influenza (A/B)	1 (2.0)	4 (7.7)	1 (3.8)	4 (14.8)	0 (0)	0 (0)
Parainfluenza virus (1–4)	15 (29.4)	15 (28.8)	8 (30.8)	9 (33.3)	7 (28.0)	6 (24.0)
Human metapneumovirus	0	1 (1.9)	0 (0)	1 (3.7)	0 (0)	0 (0)

Numbers are presented with percentages in parentheses

Respiratory symptoms had resolved in 34 of 52 (65.4%) children when sampled at week 8. All these asymptomatic children still had one or more viruses detected at week 8, with HRV detected in 91.2% (31/34) and AdV in 61.8% (22/34). Multiple viruses were detected in 20.6% (7/34) of asymptomatic children (Supplementary Table 1).

## Discussion

To our knowledge this is the first study investigating the prevalence of respiratory viruses in children presenting with suspected PTB and documenting the presence of viruses at presentation and during follow-up. We found a very high prevalence of respiratory viruses in all children, with HRV and AdV being the most frequently detected. The burden of respiratory viruses remained high at follow up, showing persistent high viral carriage. There was no clear association between viruses and TB disease categories, but HRV and multiple virus detections were more frequently seen in TB cases compared to ill controls.

Although there is limited paediatric data on what constitutes a protective immune response after *M.tb* exposure, a predominant T helper 1 response is considered protective, while a T helper 2 response is associated with severe and disseminated TB disease [13, 14]. We speculate that concurrent or sequential infections of *M.tb* and respiratory viruses may lead to an altered host immune response, which may result in inadequate containment of *M.tb* infection [15]. In support of this hypothesis, one well-studied example is influenza virus infection which often is associated with subsequent bacterial pneumonia [16]. This increased susceptibility to secondary bacterial infections and decreased bacterial clearance has been attributed to the deleterious effects of viruses on innate immunity [16–19]. Evidence from mouse models suggests that respiratory viruses such as influenza also impair the development of acquired immunity against *M.tb,* resulting in decreased protection and ability to control disease [20, 21]. Likewise, *M.tb* infection may result in a decreased immune response against subsequent viral infections. Viral infections may lead to exacerbation of TB disease and/or reactivation of TB infection [20, 21]. Although there are no data available on viral clearance in children with PTB, several studies have shown prolonged detection of viruses in individuals living with HIV or with lung conditions such as asthma or cystic fibrosis, compared to healthy controls [22–24]. In order to unravel the complex relationship between *M.tb* and respiratory viruses, future work should include investigation of organism-specific immune responses.

The literature on respiratory co-infections in children with TB is limited. The only comparable study of children with suspected PTB in South Africa was by Dube and colleagues [5]. Overall, the authors detected viruses less frequently than in our study. HMPV was the most prevalent virus detected in that study, in 19% of all the children, compared to < 2% HMPV prevalence in our study population, whereas HRV was detected in 15% of children, compared to 74% in our study. Both studies were conducted over the course of a complete calendar year, making seasonal variation an unlikely explanation for the differences observed, however differences between years when the respective studies were done could partly explain this. Some notable differences between these studies include the age of the study population, the geographical location they were recruited from and the sampling method. Children in our study were younger (22 vs 36 months) and we collected NPAs and not nasopharyngeal swabs. Viral detection is generally higher in younger children due to immature immunity (leading to increased viral replication), first exposure to particular virus strains, therefore lacking adaptive immune responses and higher viral exposure at day care and crèches [25, 26]. The different sampling strategies could also explain the differences between type and burden of respiratory pathogens detected, with NPA possibly being a more appropriate sample for viral detection in children presenting with respiratory illnesses [27, 28]. Although the respiratory virus detection rate is very high in our cohort, the data are consistent with that reported in young children with respiratory illnesses globally [29–31]. In addition, the seasonal pattern seen in our study is comparable to the South African surveillance data from 2016 to 2017, reported by the National Institute for Communicable Diseases (NICD) [32]. In 2016 the NICD reported that the RSV season started in February and peaked in May, while influenza was detected from May to November [32]. A temporal relationship between influenza virus and TB has been described, with influenza activity being followed by a peak of PTB cases a few months later; however, the number of influenza virus positive samples in our study was too small to determine an association [33].

In the literature polymicrobial detections are reported in up to 30% of infants with a lower respiratory tract infection, and this has been linked to more severe clinical disease, although the mechanism behind this is not completely understood [34, 35]. In our study, we found that TB cases more often had multiple viral co-infections compared to ill controls. This could be as a result of impaired immunity to respiratory viruses due to *M.tb* infection or, conversely, the multiple viruses result in impaired immunity and an inadequate response to control TB infection. Both scenarios are plausible and will likely depend on timing of pathogen exposure (both viral and *M.tb*) and the maturity of the host immune system. However, multiple viruses were also detected in > 20% of the children without respiratory symptoms at follow-up, indicating that further data on the clinical relevance of viral detection in children are needed.

HRV has been the most frequently detected respiratory pathogen since the introduction of molecular detection techniques [36], with prevalence reaching 50% among children presenting with respiratory illnesses [37]. The clinical relevance of HRV has often been debated; historically, HRV was seen as a common cold virus [38], however recently more severe lower respiratory infections due to HRV have been described [39, 40]. We found that HRV was more often detected in TB cases (confirmed and unconfirmed) compared to ill controls. Perhaps HRV infection facilitated a progression from *M.tb* infection to disease, which is consistent with some in vitro studies showing that HRV infection impaired phagocytosis and cytokine production against bacteria in adults with chronic obstructive pulmonary disease [41]. Alternatively, HRV co-infection could have caused symptoms resulting in hospitalization and (early) unmasking/detection of TB disease. This is consistent with other studies showing more severe clinical presentation of HRV infection in children who are younger [42, 43], with lower premorbid lung function [44] or other respiratory comorbidities [22, 23, 45].

AdV was the second most prevalent pathogen in our study, detected in more than half of children, with no differences between TB diagnostic categories. This prevalence is higher than the AdV prevalence reported in other studies [5, 29–31]. Dube and colleagues found that 7% of children with suspected PTB had AdV [5]. The majority of primary AdV infections occur during the first 5 years of life, and the age difference between the two study populations could explain some of the differences in AdV detection [46]. The high burden we observed is concerning, as there is a known association between AdV pneumonia and chronic pulmonary sequelae in young children. A meta-analysis showed that more than half of children hospitalised with AdV pneumonia had subsequent respiratory sequelae, including bronchiectasis and bronchiolitis obliterans [47]. Although AdV is often associated with severe respiratory disease, a prospective study by Self and colleagues [48] showed that AdV is also frequently detected in asymptomatic children. Long-term follow up of these children and AdV typing may elucidate the role of AdV infection on clinical presentation and long-term sequelae in children with and without PTB.

The comparable frequency of respiratory virus detection in children from baseline and follow up samples is striking and to our knowledge has not previously been described. This is especially relevant because over 90% of children that were asymptomatic at follow-up still had one or more viruses detected. This could be due to an overall high burden of respiratory viruses in our population, both in the hospital setting (at enrolment) and in households/ community (during follow-up). Alternatively, respiratory viral infections are often asymptomatic and may contribute to a natural, or, in some, a disturbed, respiratory ecosystem. This high viral detection seen in our study supports the concept described by Man et al. [49], suggesting that the clinical respiratory disease phenotype is a result of an interplay between the entire respiratory microbiota and host characteristics. They were able to differentiate children with respiratory disease from healthy controls by combining viral, bacterial, and host-related predictors. It is therefore important to consider the entire respiratory ecosystem when investigating respiratory disease, rather than focussing on single pathogen aetiology.

The main limitations of this study are the small sample size and the lack of healthy controls. Given that the overall prevalence of viral infection was so high it was not possible to directly assess the impact of viral co-infection on severity of TB disease or presentation. The small numbers limit our ability to show significant differences between virus detection in TB cases and ill controls, especially regarding their role in the clinical presentation and severity of TB disease. In addition, we lack samples prior to the current presentation; it is possible that viruses were present before children presented with symptoms of TB. A strength of this study is the careful follow-up of both TB cases and ill controls and the repeated respiratory sampling to document longitudinal prevalence of respiratory viruses in young children with and without TB.

## Conclusions

In summary, respiratory viral co-infections are frequently seen in young children with suspected PTB and viruses probably play an important role in the acquisition, control and presentation of PTB disease in young children. The interaction between host immune responses, viral infections and TB is likely complex but important to unravel to improve our understanding on *M.tb* control and possible therapeutic interventions.

## Supplementary Information

**Additional file 1: Table S1.** Virus detection in children with and without respiratory symptoms at week 8.

## Data Availability

Information in our database is confidential, however, data used for the analysis is available upon reasonable request from corresponding author.

## Abbreviations

TB: Tuberculosis
PTB: Pulmonary Tuberculosis
HIV: Human immunodeficiency virus
*M.tb*: *Mycobacterium tuberculosis*
TST: Tuberculin skin test
CXR: Chest X- ray
NPA: Nasopharyngeal aspirate
AdV: Adenovirus
InfV A/B: Influenza A/B virus
PIV 1–4: Parainfluenzavirus 1–4
HRV A/B/C: human Rhinovirus A/B/C
RSV A/B: Respiratory syncytial virus A/ B
HBoV 1–4: Human Bocavirus 1–4
hMPV: Human Metapneumovirus
HCoV 229E/NL63/OC43: Human Coronavirus 229E, NL63, OC43
HEV: Human Enterovirus
NICD: National Institute for Communicable Diseases
