# Supplementary material for: High burden of viral respiratory co-infections in a cohort of children with suspected pulmonary tuberculosis
Source: BMC Infect Dis. 2020 Dec 4;20:924. doi: 10.1186/s12879-020-05653-9 (PMC7716283; doi:10.1186/s12879-020-05653-9)
Supplement: Supplementary file 1 — Additional file 1: Table S1. Virus detection in children with and without respiratory symptoms at week 8. [file 12879_2020_5653_MOESM1_ESM.docx]

**Supplemental Table 1. Virus detection in children with and without respiratory symptoms at week 8.**

| Virus detected | Symptoms at week 8 | | p-value |
| --- | --- | --- | --- |
|  | Cough and/ or wheeze  N=18/52 (34.6) | No Cough or wheeze  N=34/52 (65.4) |  |
| Human Rhinovirus | 12 (66.7) | 31 (91.2) | 0.07 |
| Adenovirus | 10 (55.6) | 21 (61.8) | 0.66 |
| Respiratory syncytial virus (A/B) | 1 (5.6) | 2 (5.9) | 1.00 |
| Influenza (A/B) | 0 (0) | 4 (11.8) | 0.33 |
| Parainfluenza virus (1-4) | 3 (16.7) | 12 (35.3) | 0.16 |
| Coronavirus (229E, OC43, NL63) | 2 (11.1) | 7 (20.6) | 0.64 |
| Bocavirus | 4 (22.2) | 12 (35.3) | 0.33 |
| Enterovirus | 9 (50.0) | 20 (58.8) | 0.54 |
| Human metapneumovirus | 0 (0) | 1 (2.9) | 1.00 |
| Multiple viruses (≥2) | 5 (27.8) | 7 (20.6) | 0.81 |

Numbers are presented with percentages in parentheses.
